# The effects of dyslipidaemia and cholesterol modulation on erythrocyte susceptibility to malaria parasite infection

**DOI:** 10.1186/s12936-019-3016-3

**Published:** 2019-11-29

**Authors:** Marion Koch, Jaimini Cegla, Ben Jones, Yuning Lu, Ziad Mallat, Andrew M. Blagborough, Fiona Angrisano, Jake Baum

**Affiliations:** 1 Department of Life Sciences, Imperial College London, Exhibition Road, South Kensington, London, SW7 2AZ UK; 2 Division of Diabetes, Endocrinology & Metabolism, Imperial College London, London, UK; 3 Division of Cardiovascular Medicine, Department of Medicine, University of Cambridge, Cambridge, UK; 4 Division of Microbiology and Parasitology, Department of Pathology, University of Cambridge, Cambridge, UK

**Keywords:** *Plasmodium falciparum*, Red blood cell, Host–parasite interactions, Flicker microscopy, Membrane biophysics, Merozoite

## Abstract

**Background:**

Malaria disease commences when blood-stage parasites, called merozoites, invade human erythrocytes. Whilst the process of invasion is traditionally seen as being entirely merozoite-driven, emerging data suggests erythrocyte biophysical properties markedly influence invasion. Cholesterol is a major determinant of cell membrane biophysical properties demanding its interrogation as a potential mediator of resistance to merozoite invasion of the erythrocyte.

**Methods:**

Biophysical measurements of erythrocyte deformability by flicker spectroscopy were used to assess changes in erythrocyte bending modulus on forced integration of cholesterol and how these artificial changes affect invasion by human *Plasmodium falciparum* merozoites. To validate these observations in a natural context, either murine *Plasmodium berghei* or human *Plasmodium falciparum* merozoites were tested for their ability to invade erythrocytes from a hypercholesterolaemic mouse model or human clinical erythrocyte samples deriving from patients with a range of serum cholesterol concentrations, respectively.

**Results:**

Erythrocyte bending modulus (a measure of deformability) was shown to be markedly affected by artificial modulation of cholesterol content and negatively correlated with merozoite invasion efficiency. In an in vitro infection context, however, erythrocytes taken from hypercholesterolaemic mice or from human clinical samples with varying serum cholesterol levels showed little difference in their susceptibility to merozoite invasion. Explaining this, membrane cholesterol levels in both mouse and human hypercholesterolaemia erythrocytes were subsequently found to be no different from matched normal serum controls.

**Conclusions:**

Based on these observations, serum cholesterol does not appear to impact on erythrocyte susceptibility to merozoite entry. Indeed, no relationship between serum cholesterol and cholesterol content of the erythrocyte is apparent. This work, nonetheless, suggests that native polymorphisms which do affect membrane lipid composition would be expected to affect parasite entry. This supports investigation of erythrocyte biophysical properties in endemic settings, which may yet identify naturally protective lipid-related polymorphisms.

## Background

Cholesterol is a key constituent of human cells and plays a key role in modulating membrane properties, influencing both membrane fluidity [1] and stiffness [2, 3]. The effect on these cellular properties is mediated by cholesterol’s flat rigid structure which is defined by the planar tetracyclic ring shape of the molecule. While an elevation in the cholesterol content contained within cell membranes is expected to lead to a reduction in cell elasticity, how these levels are regulated and how dynamic they are in human erythrocytes remains largely unknown. A significant change in plasma lipid levels is medically described as dyslipidaemia, a term used to categorize a number of conditions, including hypercholesterolaemia, which is associated with an increase in plasma cholesterol levels. Importantly, whether and how such an increase in plasma cholesterol would affect cellular membrane composition is not clear. For example, a number of studies have found differences in how treatment of elevated plasma cholesterol or clinical conditions with dyslipidaemia is associated with differences in erythrocyte membrane cholesterol levels [4–6].

Erythrocyte infection lies at the heart of all symptoms of malaria disease. It is established when the blood-stage merozoite form of the *Plasmodium* parasite attaches to and penetrates the erythrocyte with concomitant formation of a parasitophorous vacuole inside [7]. There is a detailed appreciation of the stepwise molecular events that characterize merozoite invasion [8], however, the role the erythrocyte plays in the process was, up until recently, largely overlooked [9]. There has been a growing appreciation in the past few years that parasite binding to the erythrocyte, stimulates biophysical changes in the red cell that likely facilitate entry making it energetically more favourable [10, 11]. Further, several key polymorphisms that protect against malaria infection may do so directly by modulating erythrocyte biophysical properties [12, 13]. These polymorphisms are generally associated with changes in either the erythrocyte cytoskeleton [12] or membrane surface proteins, such as components of the glycophorin family, a well-studied group of erythrocyte surface receptors that are known to be under natural selection, likely from malaria [13–15]. To date, however, there is little study of the effects of lipid changes in mediating susceptibility to invasion and whether changes in erythrocyte membrane lipid composition might be associated with changes in efficiency of malaria parasite entry. Several studies have explored the complex relationship between obesity, nutrition and malaria. Obesity has been implicated in being protective against cerebral malaria in a mouse model of malaria infection, although no significant difference in parasitaemia was recorded and the mechanism linking the two is still unclear [16]. Other studies, also in mice, have noted a correlation between malaria infection and outcome in hypoglycaemia and hyperinsulinemia models [17], as well as with mice under calorie restriction [18]. The latter study was found to be due to nutrient sensing by the parasites and subsequent adjustment of multiplication rates through changes in gene expression levels according to nutrient availability. Finally, in humans, assessment of clinical malaria cases in Nigeria noted a negative correlation between malaria infection and serum cholesterol levels [19] though the power of the study was relatively low.

Given the implied linkages between diet, cholesterol levels and malaria and the clear role cholesterol plays in defining membrane properties of cells, the effect of elevated cholesterol levels on erythrocyte biophysical properties and susceptibility to malaria parasite infection was investigated using both an in vitro human *Plasmodium falciparum* and murine *Plasmodium berghei* model.

## Methods

### Human blood samples and serum cholesterol measurements

Human erythrocytes (O+ , male) for parasite invasion work were obtained from the NHS Blood and Transplant. Approval for collection of clinical human blood samples was granted via the Imperial College Healthcare Tissue Bank, National Research Ethics approval number 17/WA/0161, project ID R18015 and all methods were performed in accordance with the stipulated guidelines and regulations. Blood samples were collected with informed consent from hypercholesterolaemic patients attending the Imperial College London NHS lipid clinic, and from severely hypercholesterolaemic patients undergoing lipoprotein apheraesis as a treatment for homozygous hypercholesterolaemia. EDTA whole blood was collected for parasite assays and parallel measurement of serum cholesterol was performed (Abbott Architect assay, North West London Pathology Blood Sciences Laboratories). Anonymized, normocholesterolaemic EDTA whole blood samples, originating from a primary care setting, were obtained from the hospital blood sciences laboratory. No samples from patients with haemoglobinopathy were included.

### Hypercholesterolaemia mouse model and serum cholesterol measurement

C57/B6 *Ldlr*^−/−^ mice were obtain directly from the Jackson Laboratory (https://www.jax.org/). The mice were fed either on normal chow (SAFE diet 105) or Western High Fat (Dietex, FAT 21%, Cholesterol 0.15%) diets for 8 weeks. Total cholesterol and HDL cholesterol were measured using an enzymatic method in a Siemens Dimensions RxL analyser, following manufacturer’s instructions.

### Cyclodextrin-complexed cholesterol

Cholesterol (Sigma-Aldrich) was made up in ethanol at a concentration of 15 mg/ml (38.8 mM). A 5% (38.8 mM) methyl-beta-cyclodextrin (MβCD) stock was made up in MQ-water and heated up on a hotplate stirrer set to 80 °C. 4 × 10 μl aliquots of the 15 mg/ml cholesterol stock was added to 400 μl of the MβCD stock on the stirrer, leaving 10 min between each aliquot. The resulting mixture contains MβCD-Cholesterol at a ratio of 10:1. The MβCD-cholesterol mixture was left stirring on the hotplate for 1 h (to evaporate the ethanol and allow complex formation) and either used immediately or stored at − 20 °C. Erythrocytes were incubated with MβCD-cholesterol complexes for 30 min at room temperature while shaking, with concentrations of up to 3.88 mM MβCD − 388 μM cholesterol. Following incubation, erythrocytes were spun down at 800×*g* and resuspended into fresh RPMI media before use for further experiments.

### Erythrocyte membrane cholesterol measurements

Membrane cholesterol was quantified using a fluorometric Cholesterol Quantitation Kit (Sigma-Aldrich, UK) according to manufacturer instructions. In short, membranes were extracted with a chloroform-isopropanol-IGEPAL CA-630 solution and spun at 13,000×*g* for 10 min, before the organic phase was transferred to a new tube and dried under nitrogen. Samples were put under vacuum for 30 min to remove any residual organic solvents before the lipid films were dissolved in Cholesterol Assay Buffer and vortexed until the mixture was homogenous. Fluorescence intensity was measured using a Tecan MPro 200 fluorescent plate reader (excitation: 535, emission: 587 nm).

### Parasite in vitro culture

*Plasmodium falciparum* parasites (D10-PHG [20]) were maintained in standard culture conditions [21]. Parasites were grown in human O+ erythrocytes at 4% haematocrit in complete RPMI1640-HEPES media (Sigma-Aldrich) supplemented with 0.3% l-glutamine, 0.05% hypoxanthine, 0.025% gentamicin and 0.5% Albumax II (Life Technologies). Parasites were grown at 37 °C in 1% O_2_, 5% CO_2_ in N_2_ and D10-PHG cultures were supplemented with 25 ng/ml pyrimethamine and 5 μg/ml blasticidin (Sigma-Aldrich).

### *Plasmdoium falciparum* merozoite invasion assays

The *P. falciparum* merozoite assay was carried out according to published protocols [22, 23]. For a standard merozoite invasion assay, approximately 90 ml of 5% synchronized late stage D10-PHG (constitutively expressing green fluorescent protein (GFP)) parasites were magnetically isolated as described in [22], incubated with 10 μM of the cysteine protease inhibitor l-transepoxysuccinyl-leucylamido-(4-guanidino)butane (E64, Sigma-Aldrich) for up to 6 h under standard culture conditions until at least 50% of schizonts had developed into parasitophorous vacuole enclosed membrane structures (PEMS) [24]. To obtain free merozoites, PEMS were centrifuged at 800×*g* for 5 min (no brake) and resuspended into a minimum of 750 μl of RPMI. The concentrated PEMS-solution is then passed through a 1.2 μm Ministart syringe filter (Sartorius Stedim Biotech) to release individual merozoites.

For quantifying erythrocyte invasion rates, 25 μl of the filtered merozoite solution was added to a prepared erythrocyte suspension (10 μl of 1.5% haematocrit) in a 96 well plate and incubated at 37 C on a shaker (300–500 RPM) for 20 min. An additional sample containing an invasion inhibitor (typically 100 nM cytochalasin D) was included in each experiment and used for accurate gating of the invaded population. Parasites were stained with 100 μl of 5 μg/ml Ethidium Bromide (EtBr) for 10 min at room temperature, washed twice in 100 μl phosphate buffered saline (PBS), and resuspended into 60 μl. Invasion was quantified by flow cytometry, acquiring a total of 100,000 events per well using a BD Fortessa flow cytometer equipped with a high-throughput plate reader. Cells were excited at 488 nm and emission read between 515 and 545 nm for GFP positivity and between 600 and 620 for Ethidium Bromide positivity. New ring-stage parasites were quantified based on their GFPpositive, EtBr-low staining profile [23]. Data analysis was carried out in FlowJo v10 by gating on cells based on the side-scatter to forward-scatter profile (i.e. gating out debris), selecting single cells based on the forward-scatter width to area ratio and finally GFP and EtBr profile. Data was visualized in Prism v7 (GraphPad).

### *Plasmodium berghei* merozoite assays

Female Theiler’s Original (TO) mice, 8–10 weeks of age (Envigo, UK) were injected with 200 μl glycerol stocks of blood infected with 3% *P. berghei* (strain ANKA 507) expressing GFP. When parasitaemia reached approximately 3%, parasites were harvested by cardiac puncture and incubated overnight at 37 °C in 30 ml complete RPMI-1640 media under low oxygen conditions. The following day schizonts were purified using a magnetic separation column (MACS, Miltenyi Biotec), Purified schizonts were ruptured with a 1.2 μm Ministart syringe filter (Sartorius Stedim Biotech) to release individual merozoites and 25 μl merozoite solution was added to blood obtained from mice of interest (10 μl at 100,000 cells per μl, counted as described below) in a 96 well plate. The culture was incubated for 20 min on a shaker at 37 °C. Cells were stained and quantified as per the *P. falciparum* merozoite assay’quantification of invasion by flow cytometry’ described above.

### Flow cytometry and bead counting

The counting bead (CountBright Absolute Cell Counting Beads, Thermo-Fisher) solution was made up of 400 μl PBS, 50 μl counting bead solution and 50 μl of 1% haematocrit blood. To ensure equal number of beads across the samples, a mastermix of PBS-Counting beads was made up first, mixed well and separated into the number of samples required before the cell solution was added to the bead-mix. Analysis was carried out on a BD Fortessa flow cytometer. Cells were excited at 488 nm and emission read between 515 and 545 nm. 2000 beads were acquired per sample. Using ratiometric analyses based on the number of beads and cells acquired, the number of cells per μl of sample can be calculated. Data analysis was carried out in FlowJo v10 by gating on cells based on based on the forward-scatter and GFP negativity and beads based on their forward scatter profile and GFP positivity.

### Flicker spectroscopy

Flicker spectroscopy was carried out as previously described [10]. Membrane oscillation recordings were taken on a Nikon Ti Microscope (objective lens: Nikon Plan Apo 100× 1.4 N.A oil immersion) using an OrcaFlash4.0 CMOS camera. Approximately 4500 frames were recorded at a frame rate of 150 (± 10 frames per second (fps)) and an exposure time of 1 ms. Data analysis was carried out using a custom-built LabVIEW (National Instruments) program that detects and extracts membrane contours. Fourier transforming gives a fluctuation power spectrum of mean square mode amplitudes hh2(qx, y = 0) as a function of mode wavenumber (qx). From these data, the bending modulus (k) and tension (s) can be fitted using the following equation:

$$h\left( {q_{x} ,y = 0} \right)^{2} = \frac{1}{L}\frac{{k_{B} T}}{2\sigma }\left( {\frac{1}{{q_{x} }} - \frac{1}{{\sqrt {\frac{\sigma }{{k_{c} }} + q_{x}^{2} } }}} \right)$$

where k_B_ is the Boltzmann constant, T is temperature, and L is mean circumference of the cell contour [10]. Examples of the full spectra and fitted spectra for human and mouse erythrocytes are shown in Additional file 1: Fig. S1.

## Results

### Artificial incorporation of membrane cholesterol inhibits *Plasmodium falciparum* merozoite invasion

**Fig. 1:**
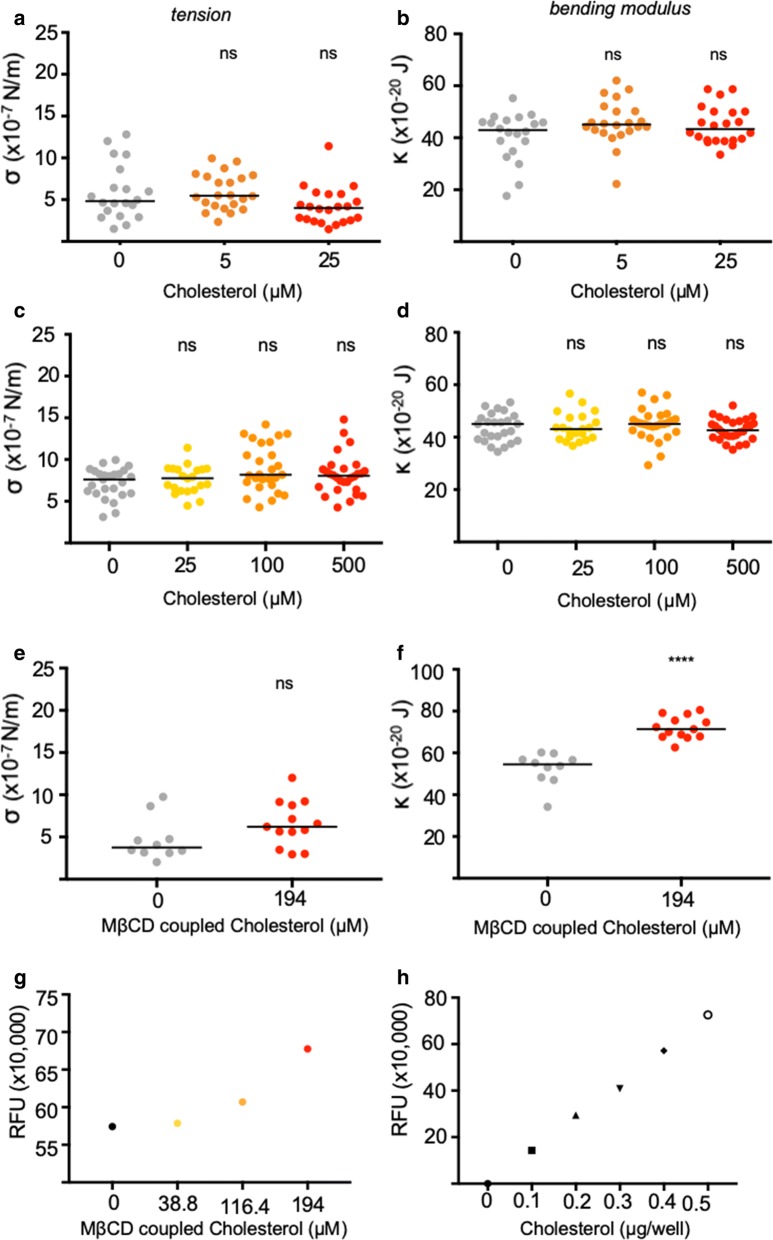
Incubating red cells with MβCD coupled cholesterol leads to incorporation of cholesterol into red cell membranes and increased red cell bending modulus. Repeated attempts to increase red cell cholesterol by incubating cells in media supplemented with cholesterol alone did not result in changes to red cell biophysical properties. Summary of (**a**, **c**) tension (σ) and (**b**, **d**) bending modulus (κ) values of red cells from two different donors pre-treated with a wide range of cholesterol concentrations. Pre-treatment of red cells with MβCD coupled cholesterol similarly did not affect red cell (**e**) tension (σ) but significantly increased red cell (**f**) bending moduli (κ). Each circle represents data from a single cell, and the solid line represents the median. To quantify the red cell cholesterol, first a cholesterol fluorometric standard curve was set up to show the linear range of the cholesterol quantitation assay (**h**). Red cells were incubated with increasing amounts of MβCD coupled cholesterol, washed and lipid membranes purified prior to cholesterol quantification. A concentration dependent increase in the cholesterol content was found in erythrocytes that had been incubated with MβCD coupled cholesterol (**g**). P values comparing the treatment versus control flicker spectroscopy data were calculated using the Mann–Whitney test (*ns* not significant; ****p < 0.0001)

**Fig. 2:**
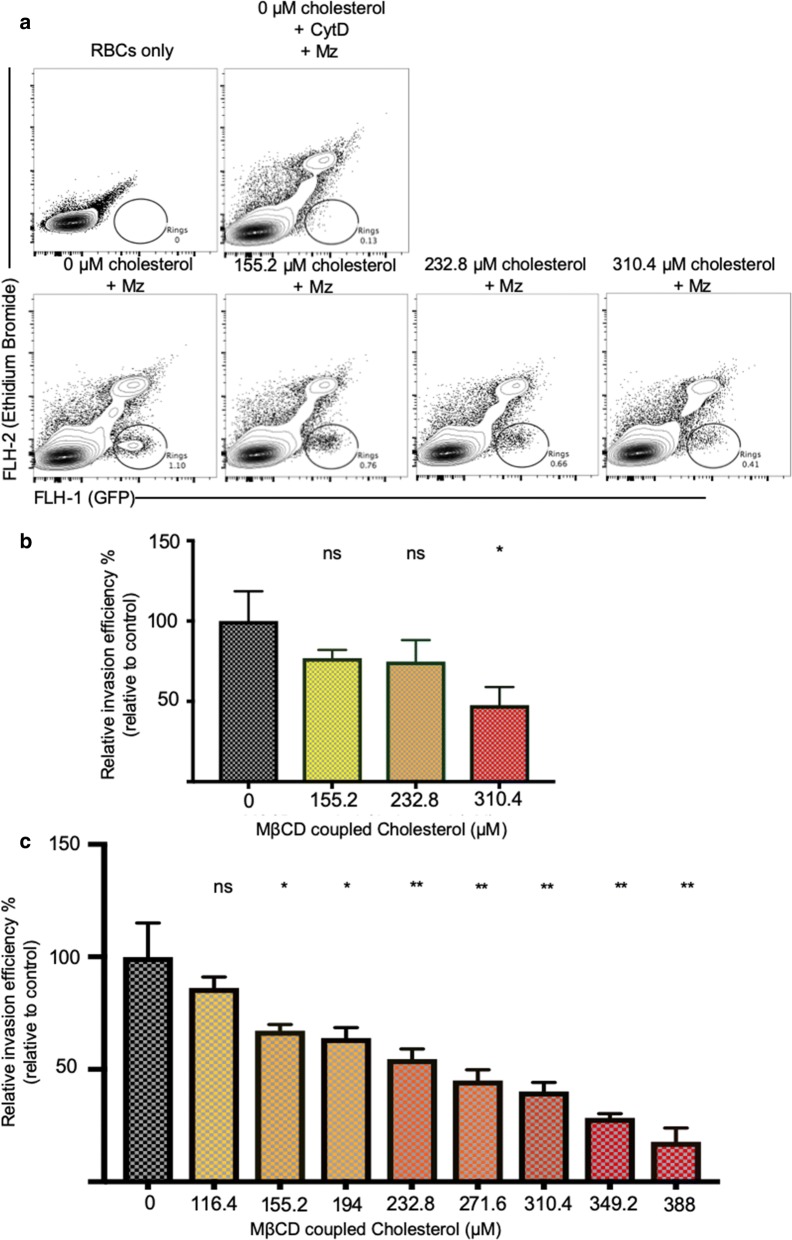
Increasing the red cell membrane cholesterol content reduces *Plasmodium falciparum* invasion efficiency. **a** Representative flow cytometry profiles highlighting infected erythrocyte populations. Top panel includes negative control (erythrocytes only), and erythrocytes incubated with merozoites in the presence of the *Plasmodium* invasion inhibitor Cytochalasin D (CytD). **b** Quantification of *P. falciparum* invasion efficiency into MβCD-cholesterol pre-treated red cells. Values represent mean and standard deviation of triplicate wells. This effect on erythrocyte invasion was reproducible across different blood donors (**c**). P values comparing the treatment versus control were calculated using the Student’s t test (*p < 0.05; **p < 0.01)

Based on previous findings, demonstrating that a reduction in the erythrocyte bending modulus resulted in an increased merozoite invasion efficiency [10], it was hypothesized that stiffening the erythrocyte membrane by increasing cholesterol content would result in a lower merozoite invasion efficiency. Erythrocytes were incubated in media supplemented with a range of cholesterol concentrations, however, no change in the red cell bending modulus was found (Fig. 1a–d). Methyl-β-cyclodextrin (MβCD) is a compound frequently used to incorporate additional cholesterol into cell membranes. Because of its high affinity for cholesterol, MβCD can be used directly to extract cholesterol from cell membranes or it can be coupled with cholesterol prior to addition to cells in order to increase cholesterol packing in the target cell membrane [25]. Thus, in an attempt to increase erythrocyte cholesterol content, red cells were incubated with MβCD coupled cholesterol. As expected, a highly significant increase in erythrocyte bending modulus was observed, whilst no significant increase in membrane tension (a measure of cytoskeletal effects [10]) was found at this concentration (Fig. 1e, f). To confirm that additional cholesterol had been incorporated into the cell membranes, cells were washed, lipid membrane extracted, purified, dried and resolubilized to measure cholesterol content. A concentration dependent increase in membrane cholesterol was observed in erythrocytes pre-incubated with MβCD coupled cholesterol (Fig. 1g). To ensure that experimental samples fell within a linear range, a cholesterol fluorometric standard curve was set up (Fig. 1h). To test whether a cholesterol-dependent increase in bending modulus is negatively correlated with merozoite invasion efficiency, erythrocytes were exposed to various concentrations of MβCD-coupled cholesterol, washed and then incubated with purified merozoites added for 30 min. Invasion efficiency was quantified using flow cytometry and showed a clear linear relationship between cholesterol-dependent increase in bending modulus and merozoite invasion (Fig. 2a–c).

### Establishing an in vitro merozoite invasion assay for *Plasmodium berghei*

**Fig. 3:**
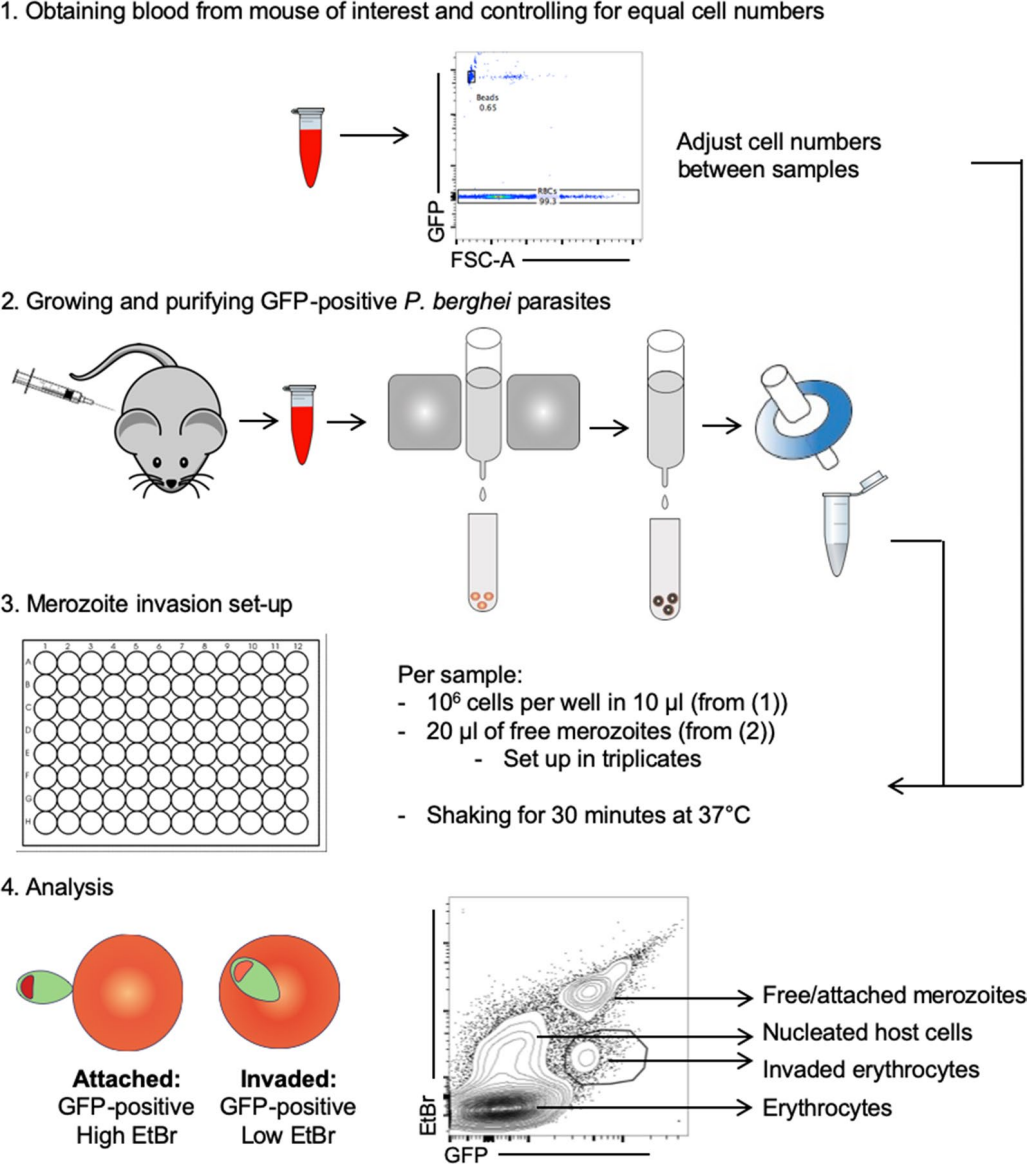
Workflow of the *Plasmodium berghei* merozoite assay. Firstly, the blood from the mouse (or mice) of interest is harvested the day prior to the invasion assay either by tail-bleed or cardiac puncture. For the assay to be quantitative, the number of cells need to be kept equal between samples obtained from different mice. This is done using a flow cytometric counting assay. Secondly, GFP-positive *P. berghei* are injected and grown in mice. At the appropriate parasitaemia, the blood is harvested by cardiac puncture 1 day prior to the invasion assay and incubated at 37 °C under low oxygen conditions until the parasites have developed into mature schizonts (12–24 h). The mature parasites are then separated from uninfected blood using a MACS magnetic cell separator, and passed through a 1.2 micrometre filter to rupture the parasitophorous vacuolar membrane and release individual merozoites [22]. Next, in a 96 well plate, three wells per mouse, each with 1 × 10^6^ blood cells, are set up before a fixed volume of free merozoites is added to each well. Invasion is allowed to occur for 30 min at 37 °C while shaking. Finally, the samples are stained with EtBr and analysed by flow cytometry. The population of erythrocytes invaded by parasites is gated on based on their GFP-positive, low EtBr staining, as the dye does not stain the intra-erythrocytic parasite as efficiently [28]

Having established a clear link between cholesterol content, erythrocyte bending modulus and *P. falciparum* merozoite invasion efficiency, the effect of increased serum cholesterol in vivo was explored to test whether it might partially protect the host from merozoite invasion using the murine malaria model *P. berghei.* Before commencing study of cholesterol effects, a workflow that can isolate invasion events in the mouse malaria model was developed. To date, study of merozoite invasion in *P. berghei* (as opposed to infection in vivo) has lagged behind that of *P. falciparum* [22] and *Plasmodium knowlesi* [26] largely because, as an in vivo model, there are few in vitro tools for its long-term propagation and, therefore, ability to isolate synchronous invasion events. Thus, to enable direct measurement of invasion (as opposed to growth) a robust workflow for merozoite isolation and invasion assessment of murine erythrocytes using *P. berghei* in vitro was established. Quantifying murine erythrocyte parasitaemia usually uses flow cytometry based on the nuclear staining of infected cells. The presence of different nucleated cell populations in mouse blood (Howell-Jolly bodies, red cell progenitors as well as leukocytes) necessitates use of a combination of antibodies and stains to allow identification of infected erythrocytes [27]. A further complication arises because of the lack of synchronicity of the parasites in vivo, with invasion occurring over a course of several hours—which can vary between individual animals—making comparison difficult. To overcome these limitations, the in vitro method for quantifying *P. falciparum* merozoite invasion [22] to *P. berghei* was adapted (Fig. 3). Using green fluorescent protein (GFP)-positive *P. berghei* isolation of synchronous parasites could be ensured that, on maturation, can be magnet separated and 1.2 μm filtered to release individual infectious merozoites. Using a 96 well-plate format a fixed volume of free merozoites were added to 10^6^ erythrocytes, allowing for 30 min invasion at 37 °C while shaking. Staining samples with ethidium bromide (EtBr), samples could then be analysed by flow cytometry. This workflow enabled quantification of an invaded erythrocyte population, which could be gated on the basis of its GFP-positive and low EtBr staining (as the dye does not stain the intra-erythrocytic parasite as efficiently [28]) (Fig. 3).

### *Plasmodium berghei* merozoite invasion efficiency is not affected by increased serum cholesterol in a mouse model of hypercholesterolaemia

**Fig. 4:**
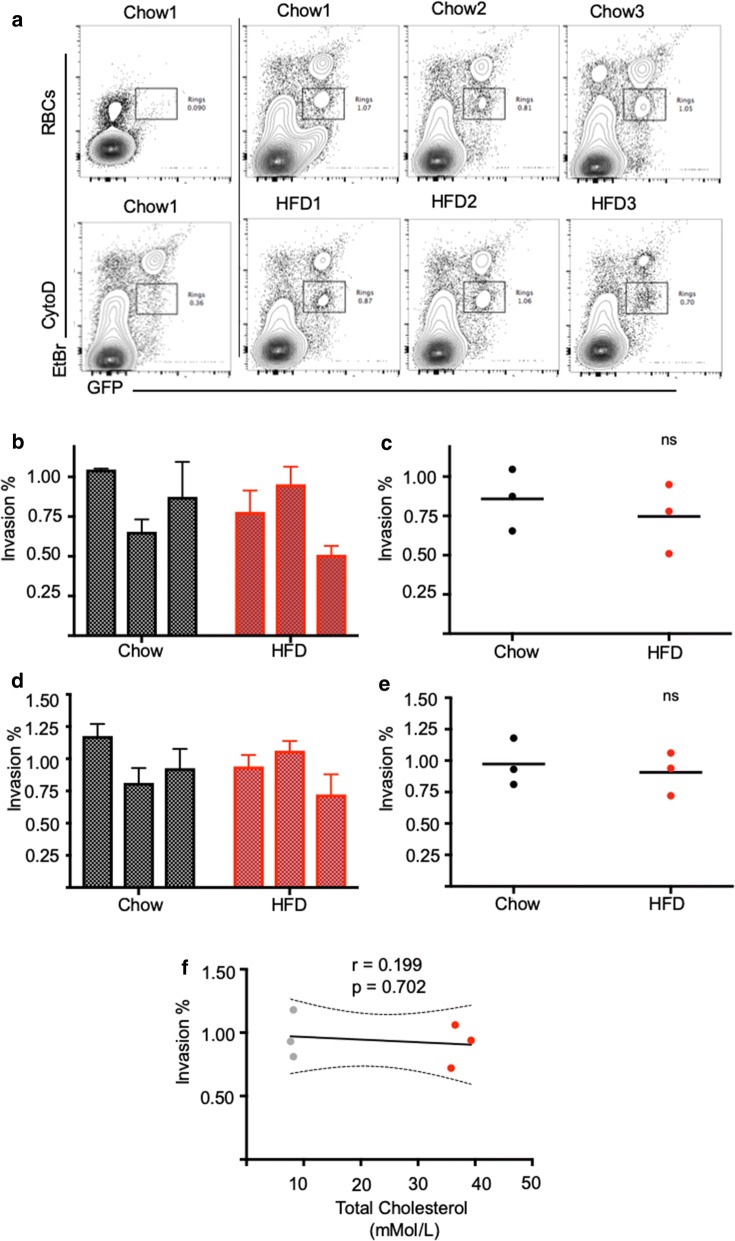
*Plasmodium berghei* invasion rates obtained from Chow and High-Fat Diet (HFD) LDL-R^−/−^ mice do not differ significantly between chow fed and high fat diets. Invasion rates obtained from Chow and High-Fat Diet (HFD) fed LDL-R^−/−^. **a** Representative flow cytometry profiles highlighting infected red cell populations obtained from mice fed the standard Chow diet (Chow) and high fat diet (HFD). A blood sample in the absence of merozoites and a sample with merozoites and CytD are shown as gating controls. (**b**, **d**) Quantification of *P. berghei* invasion assays carried out on two separate days with newly harvested parasites obtained from different mice. Values represent mean and standard deviation of triplicate wells. The invasion percentages summarized per group are shown for both experiments in (**c**, **e**). Each circle represents the average invasion percentage per sample, line represents mean. No significant differences were found in erythrocyte invasion efficiency between Chow and HFD groups. The correlation between red cell invasion and total plasma cholesterol is shown in (**f**) along with the Pearson correlation coefficient (r), p value and a linear regression line modelling the correlation with 95% confidence intervals (CI). The correlation is not significant (p > 0.05). P values comparing *P. berghei* invasion into red cells from HFD or Chow fed mice were calculated using the Student’s t test (*ns* not significant)

To test whether hypercholesterolaemia does indeed stiffen the erythrocyte membrane and provides some protection against *P. berghei* merozoite invasion, an appropriate mouse model of hypercholesterolaemia was sought. Unlike humans, mice do not efficiently absorb excess cholesterol through diet and are therefore protected from hypercholesterolaemia [29] without perturbation of cholesterol metabolism. Several genetically modified mouse models have been developed to overcome this issue; by far the most commonly used are Low Density Lipoprotein (LDL) receptor knockout (LDL-R^−/−^) and ApoE knockout (ApoE^−/−^) strains [30]. Perturbations of either pathway disrupt reverse cholesterol transport leading to excess LDL cholesterol in the circulation. *P. berghei* merozoite invasion was tested in the hypercholesterolaemia susceptible mouse strains LDL-R^−/−^. Six mice were used, three placed on a standard diet with the other three placed on a high fat diet for 8 weeks before blood was harvested by cardiac puncture and used for quantitative merozoite invasion assays. Using two independent parasite cultures on separate days, no significant difference was found in merozoite invasion efficiency between the standard and high-fat diet groups (Fig. 4a–e). Thus, although the sample size was small, no correlation was found between merozoite invasion efficiency and total plasma cholesterol (Fig. 4f).

### Erythrocytes from mice with high serum cholesterol do not show altered biophysical properties

**Fig. 5:**
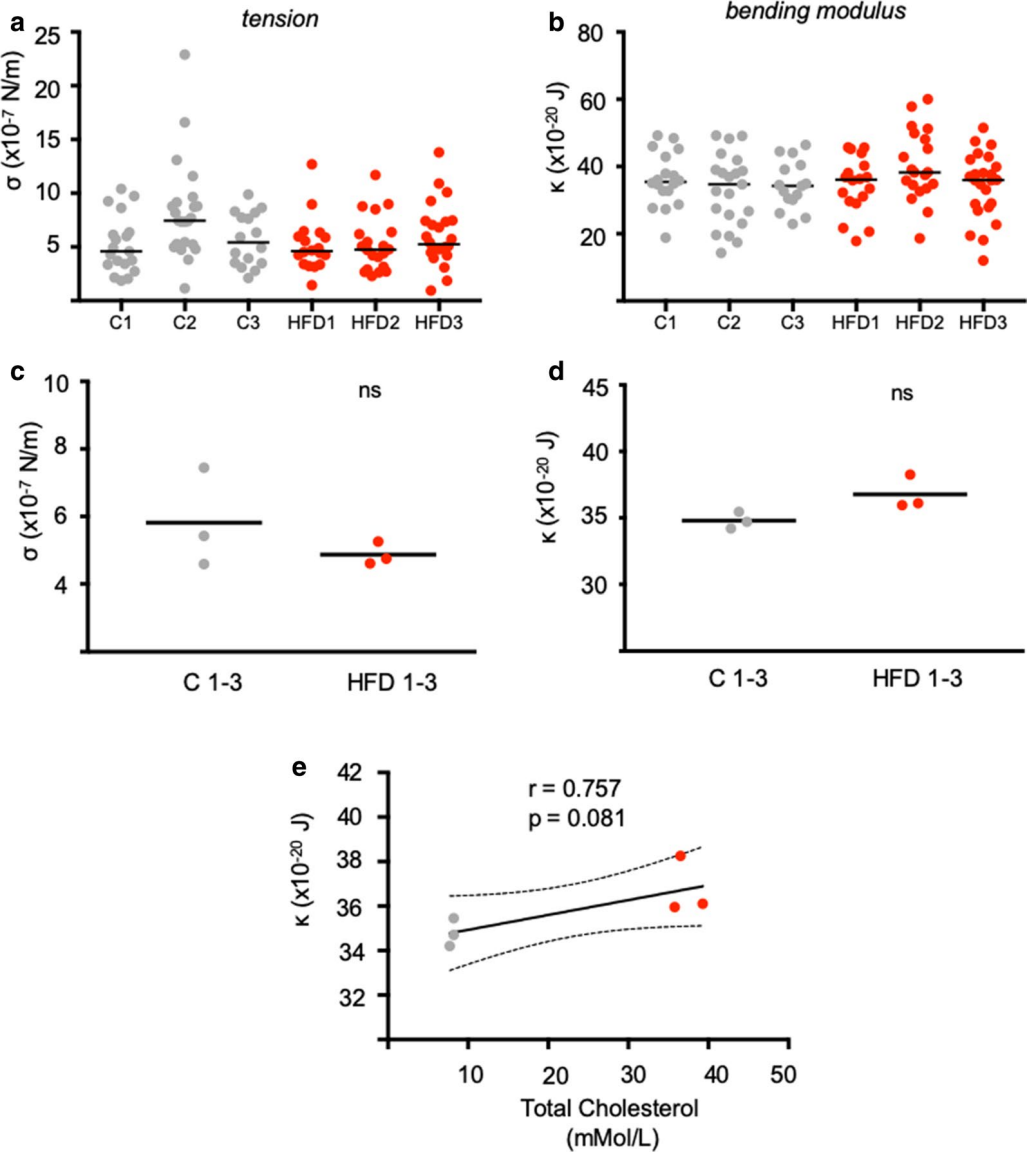
No significant changes in red cell biophysical properties were found between cells obtained from Chow or HFD-fed LDL-R(−/−) mice. Summary of tension (**a**) and bending modulus (**b**) values measured using flicker spectroscopy. Each circle represents data from a single cell, the solid line represents the median. Inter-group comparison of Chow and HFD erythrocyte tension (**c**) and bending modulus values (**d**) revealed no significant differences between the groups. Each dot represents the median tension and bending modulus obtained in **a**, **b**, solid line represents the mean. The correlation between bending modulus (κ) and total plasma cholesterol is shown in **e** along with the Pearson correlation coefficient (r), p value and a linear regression line modelling the correlation with 95% confidence intervals (CI). The correlation is not significant (p > 0.05). P values comparing the HFD versus control were calculated using the Student’s t test (*ns* not significant)

To explore why serum cholesterol did not correlate with invasion efficiency, as it had in vitro with *P. falciparum* merozoites into erythrocytes with artificially elevated membrane cholesterol, the effect of high fat diet was tested to see whether it resulted in increased serum cholesterol levels and stiffer erythrocyte membranes in LDL-R^−/−^ mice. Plasma cholesterol was quantified and the erythrocyte bending modulus was measured using flicker spectroscopy. The plasma cholesterol and triglyceride levels of LDL-R^−/−^ mice are summarized in Table 1. Plasma cholesterol levels of mice fed the high fat diet were significantly higher (p < 0.0001) than mice fed the standard diet as expected. Both HDL and LDL levels were elevated, however the biggest increase was observed in the LDL-cholesterol levels. A minimum of 20 erythrocytes from each Chow fed and High Fat Diet (HFD) LDL-R^−/−^ mice were then analysed using flicker spectroscopy. Tension and bending modulus values of individual erythrocytes are summarized in Fig. 5a, b. Notably, the median tension levels of the erythrocytes from LDL-R^−/−^ mice were found to be comparable across all samples, ranging between 4.6 and 5.4 × 10^−7^ N/m except for Mouse C2 for which the median tension is considerably higher at 7.4 × 10^−7^ N/m (Fig. 5c). The reason for the higher erythrocyte tension in this mouse is not known. The median bending modulus values were also found to be comparable across the samples, ranging from 34.2 to 38.3 (Fig. 5d). Thus, no significant correlation was found between plasma cholesterol levels and erythrocyte bending modulus. This suggests that the large increase in the plasma cholesterol levels in the HFD fed mice does not significantly impact on the erythrocyte membrane cholesterol in mice.

**Table 1:** Plasma cholesterol quantification of LDL-R^−/−^ mice

Sample	Cholesterol (mmol/l)	Triglycerides (mmol/l)	HDL (mmol/l)	LDL (mmol/l)
Chow 1	8.2	1.6	1.65	5.8
Chow 2	8.2	1.7	1.23	6.2
Chow 3	7.7	2.4	2.56	4.0
HFD 1	39.3	4.5	2.33	34.9
HFD 2	36.5	4.5	2.44	32.0
HFD 3	35.8	4.5	2.43	31.3

### *Plasmodium falciparum* merozoite invasion efficiency is not affected by increased serum cholesterol in patients with hypercholesterolaemia

**Fig. 6:**
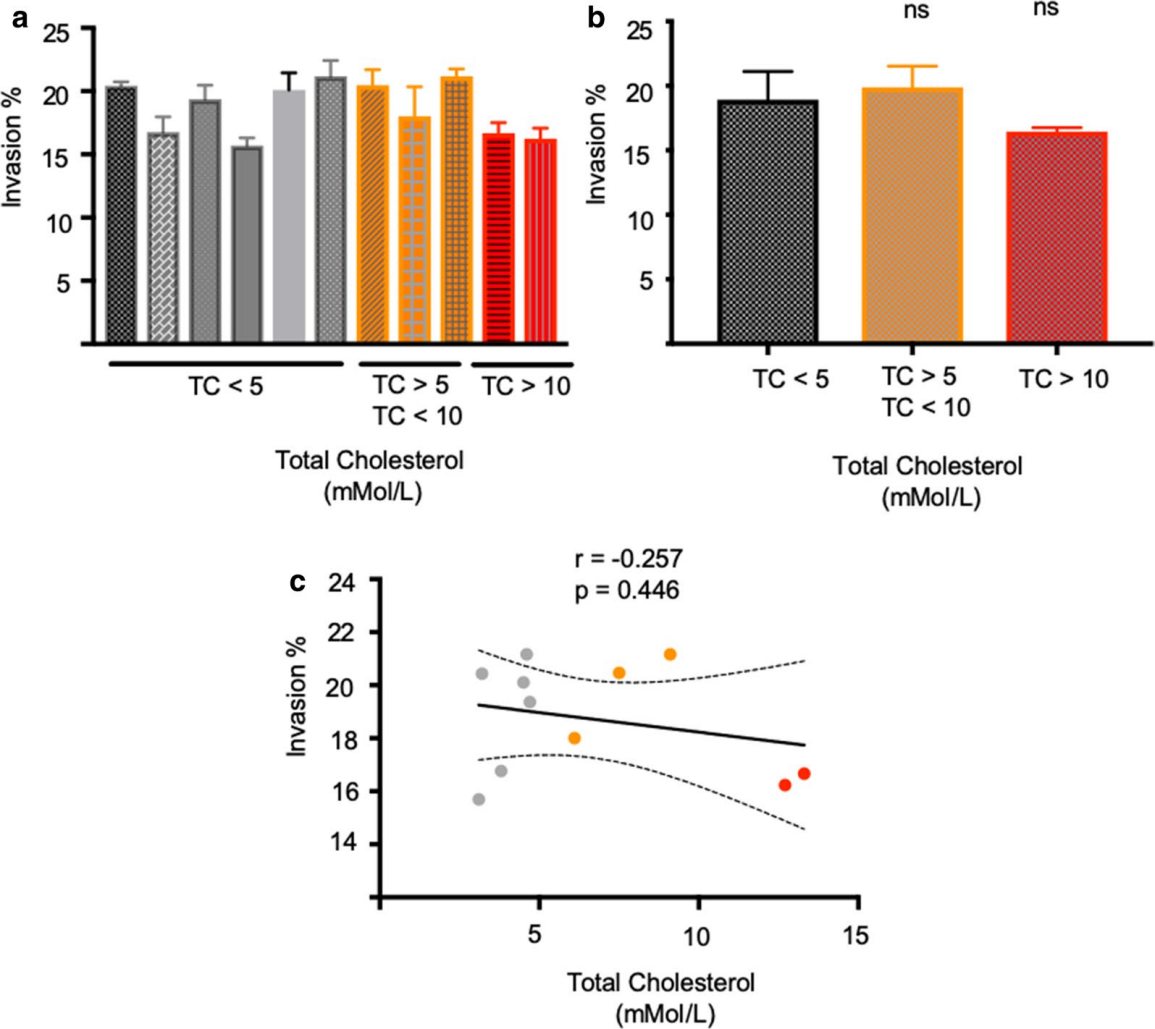
*Plasmodium falciparum* invasion rates into red cells obtained from patients with varying total plasma cholesterol (TC) levels do not differ significantly. **a** Quantification of *P. falciparum* invasion rates into red cells from patients with low TC (< 5), elevated TC (> 5, < 10) and severely elevated TC (> 10). Values represent mean and standard deviation of triplicate wells. The invasion percentages summarized per group are shown for both experiments in **b**. The correlation between red cell invasion and total plasma cholesterol is shown in **c** along with the Pearson correlation coefficient (r), p value and a linear regression line modelling the correlation with 95% confidence intervals (CI). The correlation is not significant (p > 0.05). P values comparing *P. falciparum* invasion into red cells from low, elevated and highly elevated TC samples were calculated using the Student’s t test (*ns* not significant)

Since metabolism in mice differs from humans in a number of ways as discussed above, human blood samples from patients with a range of cholesterol levels were collected and tested towards linking plasma serum cholesterol and merozoite invasion efficiency. Blood samples from patients with normal cholesterol levels (up to 5 mmol/l) were compared with samples from patients with heterozygous familial hypercholesterolaemia (elevated levels: between 5 and 10 mmol/l) as well as samples from patients with homozygous familial hypercholesterolaemia (severely elevated cholesterol levels: above 10 mmol/l). Strikingly, and mimicking that found with mouse models, the level of serum cholesterol again had no significant impact on *P. falciparum* merozoite invasion efficiency (Fig. 6a–c).

### Erythrocytes from patients with high serum cholesterol do not show altered biophysical properties or cholesterol content

**Fig. 7:**
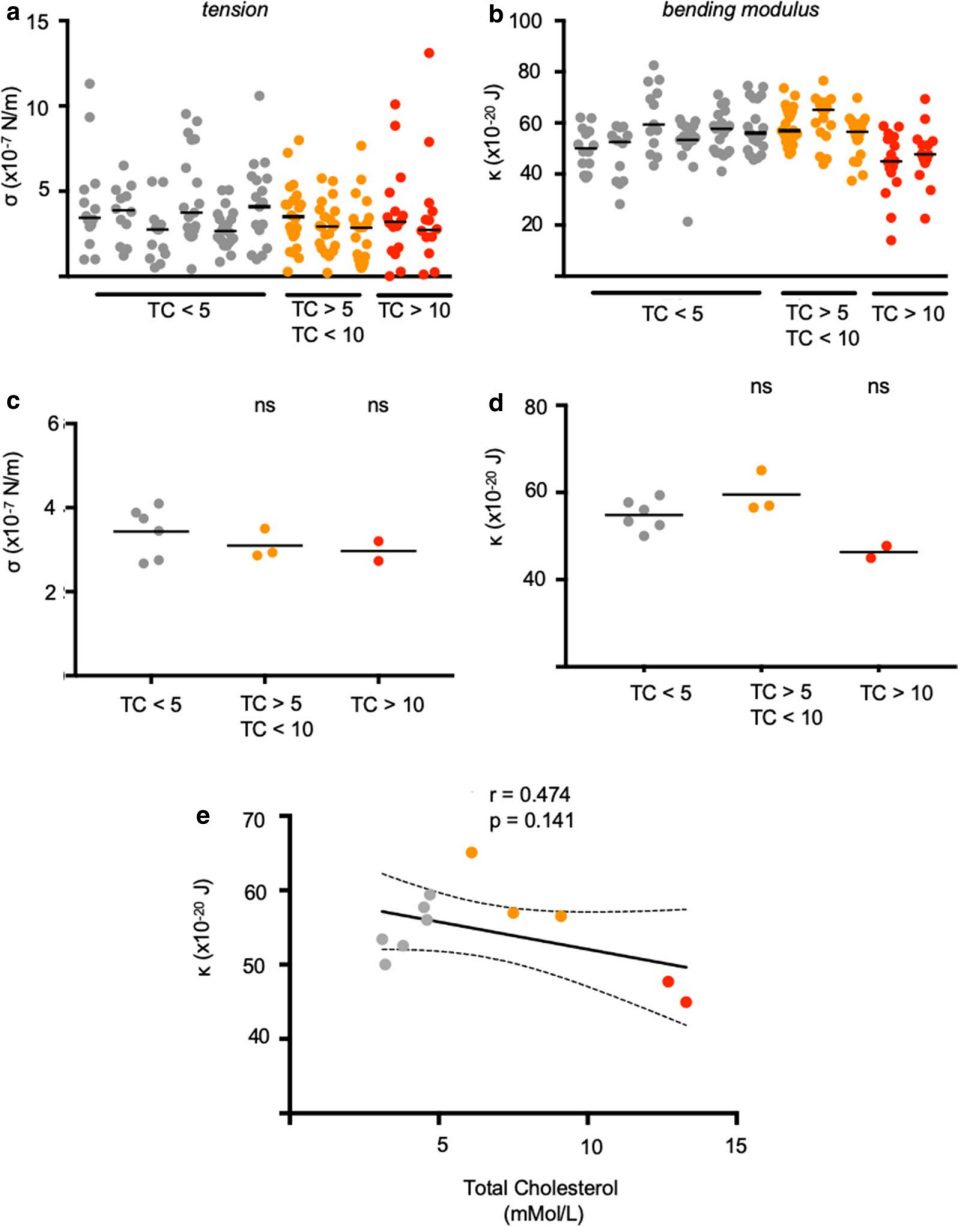
No significant changes in red cell biophysical properties were found between cells obtained from patients with varying total plasma cholesterol (TC) levels. Summary of tension (**a**) and bending modulus (**b**) values measured using flicker spectroscopy. Each circle represents data from a single cell, the solid line represents the median. Inter-group comparison of low TC, elevated and highly elevated TC red cell tension (**c**) and bending modulus values (**d**) revealed no significant differences between the groups. Each dot represents the median tension and bending modulus obtained in **a**, **b**, solid line represents the mean. The correlation between bending modulus (κ) and total plasma cholesterol is shown in **e** along with the Pearson correlation coefficient (r), p value and a linear regression line modelling the correlation with 95% confidence intervals (CI). The correlation is not significant (p > 0.05). P values comparing the HFD versus control were calculated using the Student’s t test

**Fig. 8:**
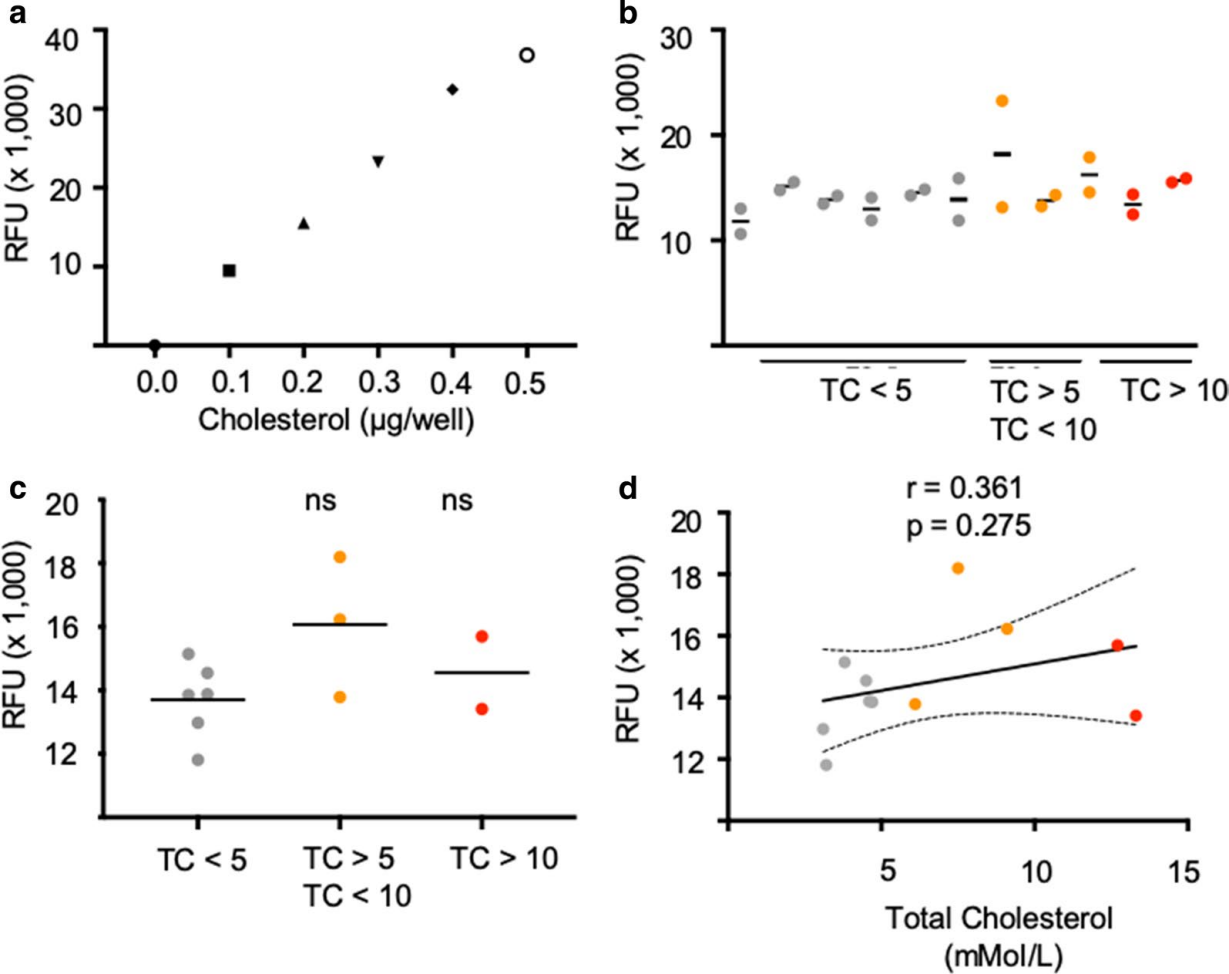
Plasma cholesterol is not correlated with red cell membrane cholesterol. **a** Fluorometric cholesterol standard curve. **b** Red cell membranes from patients with low, elevated and highly elevated total plasma cholesterol were extracted and dried before being quantified using the Cholesterol Quantitation kit (Sigma). **c** Inter-group comparison of cholesterol membrane measurements revealed no significant differences between the low, medium and high plasma/total cholesterol groups, solid line represents the mean. The correlation between membrane cholesterol and total plasma cholesterol is shown in **d** along with the Pearson correlation coefficient (r), p value and a linear regression line modelling the correlation with 95% confidence intervals (CI). The correlation is not significant (p > 0.05). P values comparing membrane cholesterol between patient samples were calculated using the Student’s t test. *RFU* Relative fluorescence units

To parallel our studies with murine erythrocytes, the effects of hypercholesterolaemia on erythrocyte biophysical properties were investigated, tension and bending modulus values for individual red cells from each patient sample (Fig. 7a, b), with group summaries shown in Fig. 7c, d. Again, paralleling mouse studies, no significant correlation was found between total serum cholesterol and red cell bending moduli (Fig. 7e). Finally, to investigate the relationship between plasma cholesterol and erythrocyte membrane cholesterol, red cell membranes from each clinical sample were dried, purified and quantified (Fig. 8a, c). No significant correlation was found between erythrocyte membrane cholesterol and plasma serum cholesterol (Fig. 8d). Thus, despite serum cholesterol levels above 10 mmol/l, it appears that human erythrocytes (and likely murine erythrocytes) are naturally buffered from incorporation of excess cholesterol. This would suggest that whilst cholesterol content of the erythrocyte is a direct correlate of malaria parasite invasion efficiency, this is not affected by plasma cholesterol levels.

## Discussion

This work set out to explore the role of erythrocyte lipid content and susceptibility to malaria. A key finding from the work is that artificial manipulation of erythrocyte cholesterol content clearly affects both red cell biophysics and merozoite invasion. By altering the cholesterol content in vitro, the erythrocyte bending modulus (a measure of membrane deformability) can be shown to markedly increase and this negatively correlates with merozoite invasion efficiency. Extending this observation to medical conditions associated with increased cholesterol levels, the question was then asked whether in vivo conditions also lead to stiffer erythrocytes and an associated reduction in merozoite invasion efficiency. Specifically, the biophysical properties of cells obtained from a murine hypercholesterolaemia model and from human clinical samples with reported hypercholesterolaemia were quantified and subsequently used in quantitative merozoite invasion assays. Strikingly, in both the murine model of hypercholesterolaemia and clinical samples of patients with elevated serum cholesterol, the elevated levels of cholesterol did not correlate with changes in erythrocyte cholesterol content. In line with this, hypercholesterolaemic blood samples showed no comparable changes in malaria parasite infection rates compared to matched controls. Thus, whilst changes in cholesterol do directly impact on red cell susceptibility to malaria parasite invasion, the balance of lipid homeostasis in the body likely buffers the red cell from altering its cholesterol content, even in the dramatic situation of hypercholesterolaemia.

Due to the difficulty in obtaining large numbers of fresh blood samples from hypercholesterolaemic patients not on statins, it is possible that very subtle effects on red cell cholesterol content may not be found with the given sample size. Investigating potential effects on erythrocyte membrane cholesterol content within a clinical setting matched the situation found in a mouse hypercholesterolaemia model, suggesting that in vivo buffering of red cell cholesterol does occur. Of note, blood samples of patients with homozygous familial hypercholesterolaemia were included, a rare condition which leads to extremely elevated levels of serum cholesterol. Even in these rare cases, red cell membrane cholesterol did not differ significantly from samples obtained from healthy patients. This therefore implies cholesterol content in the erythrocyte is tightly regulated and not significantly affected by serum cholesterol content.

The lack of correlation between plasma and erythrocyte membrane cholesterol was surprising considering that a similar study carried out in two animal models of hypercholesterolaemia found both plasma and membrane cholesterol levels responding to diet [31], suggesting that erythrocyte membrane cholesterol is dynamic and modifiable under certain conditions. In humans, the picture is not as clear, while both diet [4] and lipid lowering drugs [6] have been shown to affect erythrocyte cholesterol content, other studies report no direct correlation between plasma and cell membrane cholesterol content [5, 32]. Importantly, how erythrocyte cholesterol content is regulated is not well understood, since erythrocytes neither produce cholesterol de novo nor contain receptors, such as LDL-R, which allow uptake of lipoprotein molecules [33]. The most likely mechanism for the induced changes in erythrocyte cholesterol content is a dynamic exchange between plasma and membrane cholesterol [34, 35], with the rate of exchange likely being influenced by the structure and composition of the cell [35].

Despite the lack of changes seen in the samples tested, the question of lipid modulation, its concomitant change in erythrocyte biophysical properties and invasion susceptibility is still pertinent. Manipulating the erythrocyte modulus using different methods (using 7KC in a previous study and cholesterol enrichment here) both affected *Plasmodium* parasite invasion efficiency. A consistent working hypothesis is that this results in a direct change in the cell’s bending modulus that significantly alters parasite invasion susceptibility. Secondary effects or interactions triggered by the induced enrichment of membrane cholesterol cannot be excluded with full certainty. Cholesterol content has been reported to influence numerous cellular processes for instance changing ligand binding activity through triggering of conformational changes in other lipid groups [36] and altering the membrane dipole potential, thereby affecting protein activity [37].

## Conclusions

The work presented here demonstrates that erythrocyte biophysical properties are markedly affected by direct modulation of lipid, here cholesterol, content. Modulating the biophysical properties in this way negatively impacts merozoite invasion efficiency. Counterintuitively, however, an increase in serum cholesterol in vivo does not significantly affect erythrocyte membrane cholesterol. Thus, classical conditions in which serum cholesterol is markedly elevated, such as hypercholesterolaemia, are not protective directly against the process of merozoite invasion. The impact of lipid changes to the erythrocyte is nonetheless suggestive that other metabolic causes of variation in erythrocyte biophysical properties caused by lipid content would still be possible. Future work focusing on sampling and identifying individuals from malaria endemic regions with erythrocytes that have abnormal biophysical properties would be predicted to enable the identification of new protective polymorphisms against malaria disease.

## Supplementary information

**Additional file 1: Fig. S1.** Flicker spectroscopy spectra of human and mouse erythrocytes. (**A**) A full fluctuation spectrum visualizing height and frequency of membrane oscillations of a human erythrocyte. Higher mode numbers are affected by noise. (**B**) Modes 5–20 (in red) is the fitted range used to extract tension and bending modulus parameters. A full (**C**) and (**D**) fitted (modes 3–18 highlighted in red) mouse erythrocyte fluctuation spectrum.

## Data Availability

All raw data is available on request.

## Abbreviations

MβCD: methyl-β-cyclodextrin
GFP: green fluorescent protein
EtBr: ethidium bromide
LDL-R: low density lipoprotein (LDL) receptor
ApoE: apolipoprotein E
HFD: high fat diet
