# Supplementary figures and images for: The effects of dyslipidaemia and cholesterol modulation on erythrocyte susceptibility to malaria parasite infection
Source: Malar J. 2019 Nov 29;18:381. doi: 10.1186/s12936-019-3016-3 (PMC6884832; doi:10.1186/s12936-019-3016-3)

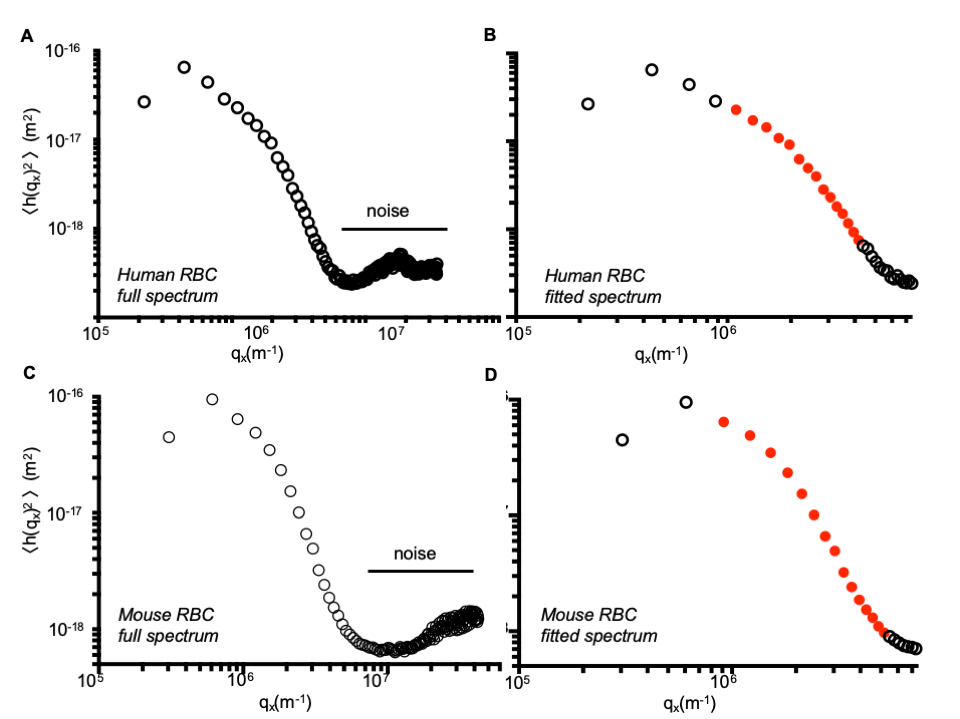

Supplement: Supplementary file 1 — Additional file 1: Fig. S1. Flicker spectroscopy spectra of human and mouse erythrocytes. (A) A full fluctuation spectrum visualizing height and frequency of membrane oscillations of a human erythrocyte. Higher mode numbers are affected by noise. (B) Modes 5–20 (in red) is the fitted range used to extract tension and bending modulus parameters. A full (C) and (D) fitted (modes 3–18 highlighted in red) mouse erythrocyte fluctuation spectrum. [file 12936_2019_3016_MOESM1_ESM.tiff]
